# The power of laughter: a study on humor and creativity in undergraduate nursing education in Egypt

**DOI:** 10.1186/s12912-024-01913-0

**Published:** 2024-04-22

**Authors:** Mona Metwally El-Sayed, Eman Sameh AbdElhay, Manal Mohammed Hawash, Samah Mohamed Taha

**Affiliations:** 1 Psychiatric and Mental Health Nursing, College of Nursing, Alexandria University, Alexandria, Egypt; 2 Psychiatric and Mental Health Nursing, College of Nursing, Mansoura University, Mansoura, Egypt; 3 Gerontological Nursing, Faculty of Nursing, Alexandria University, Alexandria, Egypt

**Keywords:** Humor, Creativity, Undergraduate students, Nursing education

## Abstract

**Background:**

Creativity in nursing education is crucial for developing practical problem-solving skills, and humor is a valuable tool for stress management and fostering a positive learning environment. This study explored the relationship between creativity and humor among nursing students.

**Methods:**

A cross-sectional survey was conducted with 265 convenient undergraduate nursing students aged 20 to 25. The Short Scale of Creative Self (SSCS) and the Multidimensional Sense of Humor Scale (MSHS) were used to measure creativity and various aspects of humor.

**Results:**

Showed a significant positive correlation between humor and creativity (*r* = 0.238, *p* < 0.001). Positive correlations were found between Humor Production, Coping or Adaptive Humor, and Appreciation of Humor with creativity (*r* = 0.254, 0.230, and 0.461, *p* < 0.001, respectively). In contrast, Attitudes Toward Humor and Humorous People were negatively correlated with creativity (*r*=-0.343, *p* < 0.05). Humor accounted for 16.2% of the variance in creativity.

**Conclusion:**

The study concluded a strong link between humor and creativity, with positive correlations observed between creative self-efficacy and creative personal identity with different aspects of humor. The study recommends the incorporation of humor into nursing education and healthcare settings to encourage creative problem-solving and reduce burnout among students and staff.

## Introduction

Nursing education gives individuals the necessary knowledge and skills to become competent healthcare providers. The American Association of Colleges of Nursing (AACN) reports that improving the educational level of nurses leads to better patient outcomes, lower mortality rates, reduced readmission rates, and shorter hospital stays [1]. Learning in nursing is usually facilitated through course materials, quizzes, assignments, and discussions. Students are encouraged to actively engage in learning and find ways to improve their understanding. However, some challenges may negatively impact their academic achievement, such as boredom [2], disinterest in the course, withdrawal, and reluctance to take responsibility [3]. Humor can create a less intimidating learning environment, increasing students’ motivation to complete their assigned activities [4–6].

Humor is a playful and light-hearted approach that can ease tension, handle uncomfortable situations, and enhance communication and comprehension in educational settings [7]. It is a complex phenomenon that involves cognitive, emotional, behavioral, psychological, and social aspects [8, 9]. It is a fundamental part of daily life and can act as a coping mechanism [8]. Humor is a positive experience that transcends cultural and social boundaries. It is the ability to recognize and appreciate the comical aspects of a situation [9]. Humor can play a vital role in engaging students in learning by directing their attention to the necessary information and creating a pleasant, emotional, and social atmosphere [10]. Humor can boost motivation in the learning process and improve students’ creative thinking abilities throughout learning activities [10].

Using humor in nursing education can be effective if it follows specific standards that ensure its positive impact. These standards include appropriateness, timing, and sensitivity to the needs of students. It is important to use humor respectfully, upholding the students’ dignity and not detracting from the seriousness of the educational environment [11]. To balance lightheartedness and professionalism, it is crucial to use humor in moderation and prevent it from becoming a distraction. Humor can be a valuable tool to break the ice, foster rapport, and reduce stress for students and educators. By applying humor thoughtfully and in line with these criteria, nursing students can have an enhanced educational experience while maintaining professionalism and respect [11, 12].

Nursing education in Egypt considers creativity a fundamental skill. Creativity is crucial in helping nurses provide top-notch patient care as nursing tasks become more complicated [13]. Creativity lies in systematically generating innovative and meaningful ideas, and it is a vital component of nursing education, encompassing the arts and sciences [14]. It is a complex and multifaceted concept with various definitions [15, 16]. Studies have demonstrated that promoting creative problem-solving skills and fostering creativity through teaching innovative teaching methods among nursing students can improve their academic performance [13, 17–19]. Therefore, it is crucial to identify the factors influencing their creativity to promote creative thinking among nursing students.

Although the research agenda stresses the significance of exploring various areas, such as personalities and impediments, in education, there has been limited empirical research on creativity in nursing education [20, 21]. However, the National Curriculum Standards in Undergraduate Nursing Program (NCSN) emphasizes that creativity, aesthetics, ethics, politics, and technical expertise are essential tools that enable students to make meaningful contributions to the care network, emphasizing patient care and a commitment to the healthcare system [22].

Chen and colleagues (2019) discovered that an excellent sense of humor is often associated with higher creative abilities. The confluence model explains the mechanism behind humor and its positive effects on creativity. From a cognitive perspective, understanding humor requires utilizing critical, creative skills. Positive emotions can lead to higher cognitive flexibility and more free associations, which benefit creativity. The emotional perspective highlights how humor fosters a positive attitude toward creativity. Appreciating humor can create positive emotions that may result in better creative output. Lastly, the motivational perspective clarifies how the happiness derived from humor leads to a strong motivation to engage in creative activities [23]. While some research indicates that positive emotions may promote unconventional ideas, other studies suggest that positive emotions may not necessarily confer any advantage in creative performance [24].

Research in nursing and education highlights the importance, relevance, and beneficial effects of incorporating humor in enhancing the educational experience [25, 26]. Humor aids in mitigating stress and fostering a sense of unity among students and faculty, creating a more supportive and cooperative atmosphere [11]. This can stimulate creativity by inspiring students to think innovatively and tackle challenges with a fresh mindset. Incorporating humor and creativity in undergraduate nursing education is indispensable for developing well-rounded and capable nurses who can adeptly handle the complexities of patient care [12]. By creating a fun and engaging learning environment, students can be more motivated and inspired to excel in their studies. This benefits the students and enhances the quality of care they can provide for their patients [25].

The significance of humor and creativity in nursing education is paramount. Integrating these concepts in nursing education is essential for nurturing a new generation of skilled, knowledgeable, compassionate, and resilient nurses. Nevertheless, there appears to be a research gap in this field, particularly in Egypt and the MENA region. This study aimed to bridge this gap by investigating the relationship between humor and creativity among nursing students to understand how humor can foster creativity and enhance nursing practices.

### Research hypothesis

Nursing students with higher humor would have higher creativity.

## Methods

### Research design

An observational cross-sectional survey, adhering to STROBE guidelines, was used, and data collection occurred from April 1st to June 30th, 2023.

### Setting

The study was conducted at the College of Nursing at Mansoura University, which is affiliated with the Ministry of Higher Education in Egypt. The college offers nine undergraduate nursing programs through a credit-hour system.

### Participants

The target population for this study was the 2nd-semester students enrolled during the 2022–2023 academic year.

### Sample size calculation

The sample size for this study was determined using specific procedures. The population data was based on the total number of registered students in the 2nd semester of 2022–2023 at the College of Nursing, Alexandria University, which was 805. The Office of Undergraduate Nursing Students provided this data. The desired precision and confidence level was set at an absolute error (d) of 5%, a type 1 error (α) of 5%, a z-score of 1.96, and a 95% confidence level, based on previous studies by Goriup et al. (2017) and Barutcu (2017) [27, 28]. The formula$\frac{\left(Z1- a/2\right)*p(1-p)}{d2}$ was used to calculate the required sample size, resulting in a minimum sample size of 238. After considering an unresponsive rate of 10%, the final sample size was adjusted to 265.

### Inclusion and exclusion criteria

The study was conducted with 2nd -semester undergraduate students from the College of Nursing at El-Mansoura University during the 2022–2023 academic year. Inclusion in the research was voluntary, and those who chose not to participate were excluded. Furthermore, students with self-reported pre-existing psychiatric conditions who received pharmacological or psychotherapies for such conditions were also excluded.

### Sampling and recruitment

**Fig. 1:**
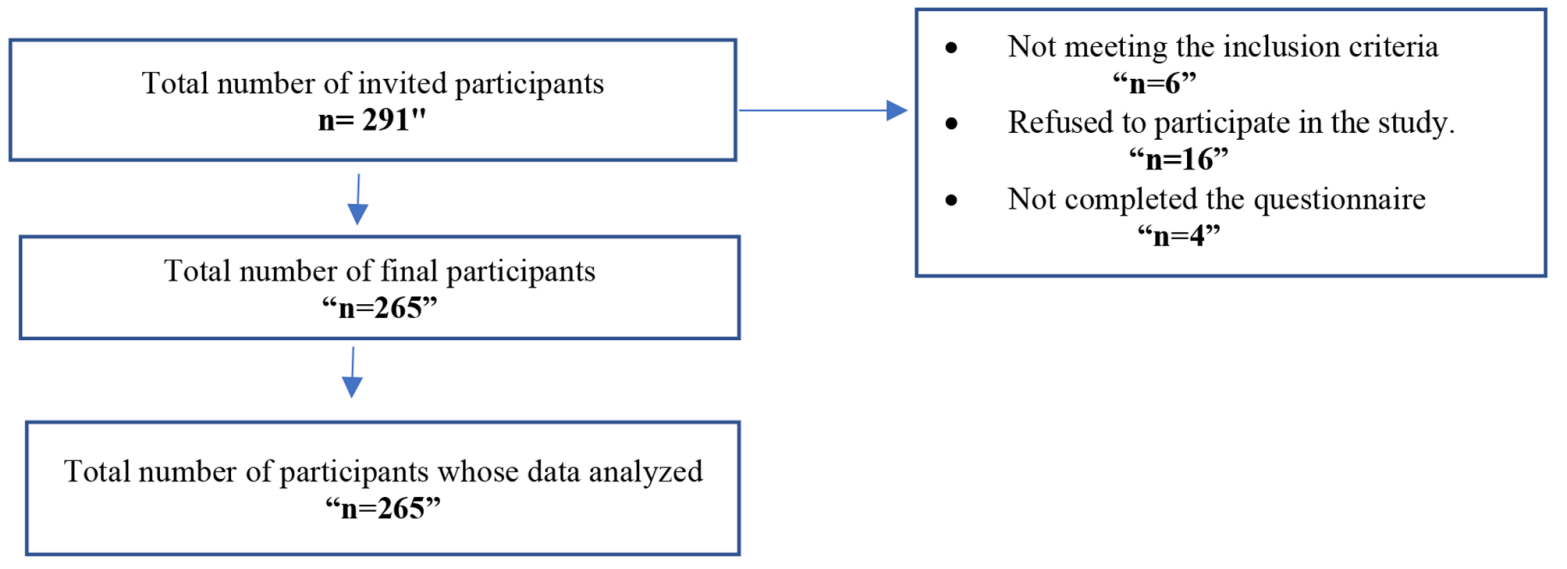
Flow chart of participants’ recruitment process

A convenient sample of undergraduate students was recruited. The total number of invited students was 291. Sixteen refused to participate, 4 initially accepted, withdrew, and did not complete the questionnaires, and 6 were deemed ineligible. These final 265 participants formed the basis for the subsequent analysis **(**Fig. 1**)**.

### Study measures

#### Demographic characteristics data sheet

The researchers developed it to elicit demographic characteristics of the participants, such as age, gender, marital status, region of residence, living arrangement, recreational activities, family monthly income, and work while studying.

### Short scale of creative self (SSCS)

The SSCS evaluates two aspects of creativity: Creative Self-Efficacy (CSE) and Creative Personal Identity (CPI) [29]. The scale consists of 11 items, with six dedicated to the CSE subscale and five to the CPI subscale. Each item is scored on a 5-point Likert scale, where 1 signifies “definitely not,” and 5 represents “definitely yes.” The total score can range from 11 to 55, with higher scores indicating greater creativity. The CSE subscale scores can range from 6 to 30, and the CPI subscale scores can range from 5 to 25. The internal consistency of the two subscales was further supported by high Cronbach’s alpha values: 0.83 for CSE and 0.89 for CPI.

### Multidimensional sense of humor scale (MSHS)

The MSHS is a comprehensive tool for assessing various aspects of humor [30]. It consists of 24 items that capture four independent dimensions of humor: (i) humor production and creativity, e.g., “Other people tell me that I say funny things.” (ii) coping or adaptive humor, e.g., “Uses of humor help to put me at ease.” (iii) appreciation of humor, e.g., “Humor helps me cope.” and (iv) attitudes toward humor and humorous people. “Calling somebody a “comedian” is a real insult”. The scale is presented as a 5-point Likert scale, ranging from 0 (strongly disagree) to 4 (strongly agree), with a possible total score between 0 and 96, calculated by summing the scores of each factor. The scale demonstrated high internal consistency, as indicated by a high Cronbach’s alpha of 0.91.

### Procedure

#### Ethical approval

Official permissions for the study were secured from the responsible authorities at the College of Nursing, Mansoura University. Before inclusion in the study, participants had to provide informed consent, which involved signing a document. The study’s purpose was communicated to the participants, emphasizing the anonymity of their responses and the assurance of confidentiality. Detailed instructions were provided on how to respond to the study tools. Utmost care was taken to respect the privacy and confidentiality of the data, which was maintained throughout the study. Students were informed of their right to participate and their freedom to withdraw from the study.

### Pilot study and reliability

Prior to the commencement of the main study, a preliminary pilot study was conducted involving 20 nursing students. These students did not participate in the main study. The pilot study confirmed that all the tools used were accurate, Comprehensible, and suitable for the study. The internal consistency of the study measures was evaluated using Cronbach’s Alpha test.

### Data collection

After the pilot study participants were excluded, a convenient sample was selected. Trained researchers conducted structured interviews with each participant, collecting necessary data using data collection tools. Each interview, lasting approximately 10–15 min, was conducted in a private setting, such as an empty classroom or clinical lab, to ensure privacy. Informed consent was obtained from each participant, ensuring their anonymity and confidentiality. The interviewers held no authority over the students, and there were no incentives for participation. The students were reassured that their participation was voluntary and that they had the right to withdraw without repercussions. All responses were kept confidential. To ensure the accuracy and completeness of the information, researchers meticulously reviewed the responses to the data collection tool provided by each participant.

### Data analysis

Data analysis was performed using IBM SPSS software (version 26.0). A meticulous review and verification were conducted following data entry to ensure accuracy. The distribution normality of quantitative variables was evaluated using Kolmogorov-Smirnov and Shapiro’s tests. Cronbach’s alpha was used to assess the internal consistency of the research instruments, thereby indicating their reliability. The humor and creativity subscales were summarized using means (M), standard deviations (SD), and frequencies or percentages for categorical variables. Pearson’s correlation coefficient measured the strength and direction of the relationship between two normally distributed quantitative variables. A multiple linear regression analysis was carried out to determine the impact of various humor domains on creativity. All results were deemed significant at the 5% (*p* < 0.05).

## Results

Table 1 shows that the majority were female (61.51%). The age distribution was even, with 32.83% being 20 or older, 28.68% between 21 and 24 years, and 38.49% being 25 years or more. Most participants were single (93.58%) and lived with their families (74.71%). The participants were almost evenly split between urban (53.20%) and rural (46.80%) residences. Regarding income, 25.66% reported their family income as insufficient, while 51.32% found it somewhat sufficient, and 23.02% considered it sufficient. A significant majority of the students (90.94%) worked while studying. Regarding recreational activities, 70.19% engaged in sports, 33.21% in art and music, 93.96% enjoyed going on trips, 26.04% participated in campus activities, and 50.57% participated in cultural activities.

**Table 1:** The demographic characteristics of the participants

-	Categories	Total (*n* = 265)	
		No	%
Gender	Male	102	38.49
	Female	163	61.51
Age	≥20	87	32.83
	21–24	76	28.68
	≥25	102	38.49
Marital Status	Single	248	93.58
	Married	17	6.42
Region of Residence	Urban	141	53.20
	Rural	124	46.80
Living Arrangement	Family	198	74.71
	Relatives	39	14.72
	Alone	28	10.57
Family Monthly Income	Not Sufficient	68	25.66
	Somewhat Sufficient	136	51.32
	Sufficient	61	23.02
Work While Studying	Yes	241	90.94
	No	24	9.16
# Recreational Activities	Doing sports	186	70.19
	Doing art and music activities	88	33.21
	Going to trips	249	93.96
	Participate in campus activities	69	26.04
	Participate in cultural activities	134	50.57

# multiple responses

Table 2 presents the participants’ average scores on the Self-Concept of Creativity Scale (SSCS) and the Multidimensional Sense of Humor Scale (MSHS). The SSCS mean score for the Creative Self-Efficacy subscale was 22.29 (SD = 4.76); for the Creative Personal Identity subscale, it was 19.31 (SD = 2.52). The total mean score for the SSCS was 41.60 (SD = 7.28). Regarding the MSHS, the mean scores were as follows: 21.48 (SD = 2.56) for the Humor Production and Creativity subscale, 14.27 (SD = 2.68) for the Coping or Adaptive Humor subscale, 7.56 (SD = 1.96) for the Appreciation of Humor subscale, and 8.58 (SD = 2.48) for the Attitudes Toward Humor and Humorous People subscale. The total mean score for the MSHS was 51.89 (SD = 9.68).

**Table 2:** The mean scores of the participants on SSCS and MSHS

	*Total* **(n = 265)**	
**SSCS**	***M***	***SD***
Creative Self-Efficacy	22.29	4.76
Creative Personal Identity	19.31	2.52
Total	41.60	7.28
**MSHS**		
Humor Production and Creativity	21.48	2.56
Coping or Adaptive Humor	14.27	2.68
Appreciation of Humor	7.56	1.96
Attitudes Toward Humor and Humorous People	8.58	2.48
Total	51.89	9.68

SSCS: Short Scale of Creative Self MSHS: Multidimensional Sense of Humor Scale

M: Mean Score SD: Standard Deviation

Table 3 presents the Pearson correlation coefficients (r) between various aspects of humor and creativity among the participants. The Creative Self-Efficacy subscale demonstrated a significant positive correlation with the Humor Production and Creativity subscale (*r* = 0.224, *p* < 0.001) and the Appreciation of Humor subscale (*r* = 0.529, *p* < 0.05). However, it negatively correlated with the Attitudes Toward Humor and Humorous People subscale (*r*=-0.224, *p* < 0.05). Similarly, the Creative Personal Identity subscale exhibited significant positive correlations with the Humor Production and Creativity subscale (*r* = 0.265, *p* < 0.001) and the Appreciation of Humor subscale (*r* = 0.417, *p* < 0.05). It also negatively correlated with the Attitudes Toward Humor and Humorous People subscale (*r*=-0.251, *p* < 0.05). The total scores of the SSCS displayed significant positive correlations with the Humor Production and Creativity subscale (*r* = 0.254, *p* < 0.001), the Coping or Adaptive Humor subscale (*r* = 0.230, *p* < 0.05), the Appreciation of Humor subscale (*r* = 0.461, *p* < 0.05), and the total of the MSHS (*r* = 0.238, *p* < 0.001). However, they had a negative correlation with the Attitudes Toward Humor and Humorous People subscale (*r*=-0.343, *p* < 0.05).

**Table 3:** The correlation coefficients (r) between various aspects of humor and creativity among the participants (*n* = 265)

Variables		Humor Production and Creativity	Coping or Adaptive Humor	Appreciation of Humor	Attitudes Toward Humor and Humorous People	Total scores of MSHS	Creative Self Efficacy	Creative Personal Identity	Total scores of SSCS
Humor Production and Creativity	***r***								
	***p***								
Coping or Adaptive Humor	***r***	0.314** 0.001							
	***p***								
Appreciation Of Humor	***r***	0.587* 0.031	0.254** 0.001						
	***p***								
Attitudes Toward Humor and Humorous People	***r***	0.419** 0.001	0.357** 0.001	0.556** 0.002					
	***p***								
Total scores of MSHS	***r***	0.316** 0.001	0.412** 0.001	0.268** 0.001	0.394** 0.001				
	***p***								
Creative Self Efficacy	***r***	0.224^**^ 0.000	0.081 0.231	0.529* 0.027	-0.224* 0.032	0.196^*^ 0.012			
	***p***								
Creative Personal Identity	***r***	0.265^**^ 0.000	0.179^*^ 0.023	0.417* 0.042	-0.251* 0.035	0.265** 0.001	0.423** 0.001		
	***p***								
Total scores of SSCS	***r***	0.254^**^ 0.001	0.230* 0.030	0.461* 0.022	-0.343* 0.021	0.238^**^ 0.001	0.214** 0.001	0.347^**^ 0.001	
	***P***								

SSCS: Short Scale of Creative Self MSHS: Multidimensional Sense of Humor Scale

r = Pearson correlation * Significant p at *P* ≤ 0.05

Table 4 presents a multiple linear regression analysis examining the effect of various aspects of humor on creativity among participants. The model was statistically significant (F = 169.782, *p* < 0.001) and explained 16.2% of the variance in creativity (Adjusted R^2 = 0.162). All four humor variables showed significant effects on creativity. Specifically, the Humor Production and Creativity subscale (B = 0.226, Beta = 0.102, *p* < 0.001) and the Coping or Adaptive Humor subscale (B = 0.071, Beta = 0.121, *p* < 0.001) had positive effects on creativity. However, the Appreciation of Humor subscale (B=-0.100, Beta = 0.075, *p* = 0.005) and Attitudes Toward Humor and Humorous People subscale (B=-0.209, Beta = 0.377, *p* < 0.001) had negative effects on creativity.

**Table 4:** A multiple linear regression analysis of the effect of humor on creativity (*n* = 265)

	^a^ MSHS		t	p	95% CI	
	***B***	***Beta***			***LL***	***UL***
^b^ Humor production and creativity	0.226	0.102	3.929^*^	< 0.001^*^	0.210	0.720
^b^ Coping or adaptive humor	0.071	0.121	5.005^*^	< 0.001^*^	0.070	0.074
^b^ Appreciation of humor	-0.100	0.075	2.801^*^	0.005^*^	-0.100	-0.269
^b^ Attitudes toward humor and Humorous people	-0.209	0.377	14.044^*^	< 0.001^*^	0.115	-0.306
R^2^ = 0.168, Adjusted R^2^ = 0.162, F = 169.782^*^, *p* < 0.001^*^						

^a^ MSHS: Multidimensional Sense of Humor Scale (independent variable) ^b^SSCS: Short Scale of Creative Self ( Dependent variable)

F, p: f and p values for the model R^2^: Coefficient of determination

B: Unstandardized Coefficients Beta: Standardized Coefficients

t: t-test of significance LL: Lower limit UL: Upper Limit

*: Statistically significant at *p* ≤ 0.05

## Discussion

Creativity is vital for nursing education, economic development, and individual well-being. The nursing profession requires creative individuals with researcher roles who have access to knowledge and can produce and use information [26]. Expanding creativity enables modern nursing applications and greater problem-solving. The current study investigated the creativity of nursing students and the elements that influence it, such as humor.

The present study revealed that nursing students possess moderate creativity, as evaluated through the SSCS, with mean scores of 22.29 and 19.31 for CSE and CPI, respectively, and a total mean of 41.60. These findings were consistent with a cross-sectional study involving 720 medical students, which found that the majority demonstrated moderate creativity. The study identified several factors that can enhance creativity, including problem-based learning, critical thinking, concept mapping, teamwork, and innovative teaching methods [31]. Another study by Qian et al. (2023) suggested that creative self-efficacy (CSE) impacts an individual’s readiness to experiment with new ideas, effort levels, and resilience when faced with challenges. Those with high CSE will likely possess sufficient psychological capital to withstand uncertainties and difficulties, leading to increased motivation, cognitive resources, and activities to meet contextual demands [32].

Along the same line, Karwowski (2016) employed a crossover longitudinal and sequential approach to examine the development of CSE and creative personal identity (CPI) beliefs over time. The study involved 976 Polish participants aged between 15 and 60 who responded to the Short Scale of Creative Self. The results indicated that these beliefs remain stable in the short term (six months), but significant changes can be observed after 20 months. The study found evidence of growth in both constructs as individuals transition from adolescence to early adulthood. However, a decrease in CPI was observed in all age groups, except those aged 15 to 24, which showed a significant increase [33]. Moreover, a focus group study explored the perspectives of medical students, postgraduate medical trainees, and medical specialists on creativity within the medical context. The study concluded that participants perceived creativity as a form of art encompassing thought and action. Creative problem-solving strategies, considered the “creative part” of critical thinking, can enhance students’ critical thinking skills. These strategies encourage students to be open-minded, curious, and reflective and think and conceptualize outside the box. This process fosters the development of their intuition, associative ability, and metaphor usage [34].

These findings could be related to the fact that in the healthcare industry, nursing students must have the requisite skills and knowledge to tackle real-life scenarios. While indispensable, clinical training can be overwhelming due to its unique challenges. From witnessing the natural progression of death to dealing with highly contagious illnesses in real time and technological advancements, these experiences can be daunting [35]. The challenges they confront necessitate the application of new concepts and abilities, prompting personal introspection and self-examination. Working with limited resources and alongside individuals with poor clinical skills forces them to learn and grow, thus becoming better prepared to handle real-life situations. Modern teaching methods of “blending learning” require self-learning, and search places a premium on creative thinking, enabling students to become innovative problem solvers.

Our research has uncovered an exciting insight - most participants scored moderate on the humor scale, with a mean score of 51.89. This finding indicates how humor is deeply ingrained in Egyptian society, where using humor to alleviate difficult situations is widely accepted [36]. Such a cultural disposition aligns with a study conducted by Jiang et al. (2019), which examined the impact of culture on the perception and usage of humor and its mental health benefits. The study found that Easterners view humor less positively than their Western counterparts. They also use humor less often as a coping mechanism, primarily due to this perception. However, despite the cultural differences, Westerners and Easterners show similar patterns in the relationship between their humor and psychological well-being scores [37]. Numerous studies have shown that incorporating humor into the therapeutic relationship can positively impact the nurse’s mental health and understanding of the patient’s care needs [36–40]. According to Chelly et al. (2022), humor helps nurses alleviate stress and anxiety while humanizing the issue. Though the context in which humor can be used is highly influenced by personal factors, such as age, gender, and personality, its use is strongly recommended. However, it is necessary to use humor cautiously and in appropriate circumstances, as there may be barriers to its use [41].

Our findings indicated that the total scores of SSCS showed a significant positive correlation with Humor Production and Creativity (*r* = 0.254), Coping or Adaptive Humor (*r* = 0.230), and Appreciation of Humor (*r* = 0.461). However, there was a negative correlation between Attitudes toward Humor and Humorous People (*r*=-0.343). Furthermore, the authors performed multiple linear regression analyses to determine the impact of various humor components on nursing students’ creativity. The model was statistically significant, accounting for 16.2% of the variance in creativity (Adjusted R^2 = 0.162). These findings could be linked to humor in educational environments, appreciated for its capacity to create a relaxed atmosphere and improve interpersonal dynamics, demonstrating humor’s diverse roles in various aspects of life. Nursing students employ humor as a coping mechanism to deal with challenges, such as imagining humorous scenarios, sharing jokes, or indulging in amusing conduct. This coping humor fosters creative problem-solving, positive emotions, resilience, and mental well-being [11, 12, 36].

These findings were congruent with another study on 228 psychological counselors aged between 23 and 52 years—of which 130 were female and 98 were male—which found a positively significant correlation between psychological resilience, psychological well-being, and coping humor. Research indicates that a sense of humor can improve physical and psychological health and overall well-being [42].

Ghayas and Malik (2013) also found that humor predicts creativity and sociability levels in university undergraduates. Moreover, humor’s creation and performance dimensions were strong predictors of creativity. In contrast, the elaboration subscale of creativity significantly predicted humor. The attitude towards humor and humorous people, along with the humor subscale, were the only significant predictors of sociability [43]. Similarly, Biemans and Huizingh (2023) employed a mixed-methods study design and found that being in a humorous mood improves creativity in specific creative situations. They highlight how research into the impact of humor on creativity and innovation can take new directions [44]. Moreover, Kocak (2018) studied the influence of various humor types on creativity and the role of university innovation environments in this relationship. The study involved 362 academics from Turkish universities. The findings revealed that aggressive humor negatively correlates with academic creativity while self-enhancing and affiliative humor positively correlate. Self-defeating humor, however, showed no significant link with creativity. The study also found that the innovation climate moderately affects the relationship between humor styles and creativity. These findings highlight the potential of different humor styles to enhance organizational productivity and creativity [45].

### Nursing implications

This study’s findings present several implications for nursing education and practice. The positive correlation between humor and creativity suggests that integrating humor-based activities and creative problem-solving training into the curriculum could enhance students’ creativity. As a valuable tool for stress management, humor could help reduce burnout among students, potentially improving their inventive problem-solving skills. Nursing educators can incorporate relevant humor into their lessons using various forms such as cartoons, spontaneous humor, role-playing, jokes, and funny stories to engage students effectively. Moreover, it could help develop more effective communication strategies, improve patient-nurse relationships, and apply innovative nursing interventions to manage health problems and enhance the overall quality of patient care. Humor can make patients feel more comfortable and open during interactions, leading to better communication and, ultimately, better health outcomes.

### Study limitations

This study offers important insights into the connection between humor and creativity in nursing education. However, it has several limitations. The findings are based on a non-probability convenient sampling technique, which may limit their applicability to a broader population. The study’s cross-sectional design prevents the establishment of a causal relationship between humor and creativity. The reliance on self-report measures could introduce social desirability bias. Future research could address these limitations using objective measurement tools, such as observational or peer-rated instruments and probability sampling methods. Longitudinal studies could provide a more nuanced understanding of how the relationship between humor and creativity evolves. Furthermore, exploring personality traits, learned helplessness, academic burnout, self-evaluation, motivation, and cultural influences as covariates with humor and creativity. Further experimental studies on humor-based activities and creative problem-solving training in nursing education to improve student creativity, stress levels, and academic performance. These studies can provide concrete evidence for the benefits of humor and creativity in healthcare settings.

### Conclusion and recommendations

Our findings underscored a significant correlation between various facets of humor and creativity. Positive correlations were observed between creative self-efficacy and creative personal identity with different aspects of humor, such as humor production, coping or adaptive humor, and appreciation of humor. Conversely, negative correlations were found with unfavorable attitudes towards humor and humorous individuals. Considering these findings, it is crucial to maintain a comfortable and humorous atmosphere in nursing education settings. Such an environment encourages creative problem-solving skills and can help reduce burnout among nursing students and staff. Therefore, it is recommended that humor be integrated into the educational system and healthcare settings to enhance creativity and overall well-being.

## Data Availability

The datasets used or analyzed in this study are available from the corresponding author upon reasonable request.

## Abbreviations

KS: Kolmogorov-Smirnov
SPSS: Statistical Package for Social Sciences
REC: Research Ethical Committee
SSCS: Short Scale of Creative Self
CSE: Creative Self-Efficacy
CPI: Creative Personal Identity
MSHS: Multidimensional Sense of Humor Scale
AACN: American Association of Colleges of Nursing
